# The prognostic impact of programmed cell death 1 and its ligand and the correlation with epithelial‐mesenchymal transition in thymic carcinoma

**DOI:** 10.1002/cam4.1943

**Published:** 2019-01-01

**Authors:** Soichiro Funaki, Yasushi Shintani, Eriko Fukui, Yoko Yamamoto, Ryu Kanzaki, Naoko Ose, Takashi Kanou, Masato Minami, Eiichi Mori, Meinoshin Okumura

**Affiliations:** ^1^ Department of General Thoracic Surgery Osaka University Graduate School of Medicine Suita‐city Japan; ^2^ Department of Pathology Osaka University Graduate School of Medicine Suita‐city Japan; ^3^ General Thoracic Surgery Toneyama National Hospital Toneyama Japan

**Keywords:** epithelial‐mesenchymal transition, induction therapy, PD‐1/PD‐L1, TGF‐β, thymic carcinoma

## Abstract

**Background:**

The significance of epithelial‐mesenchymal transition (EMT) and immune checkpoint proteins in thymic carcinoma remains unknown. We examined the clinical significance of EMT, tumor‐infiltrating lymphocytes expressing the immune checkpoint protein, programmed cell death 1 (PD‐1 + TILs), and the expression of PD‐1 ligand 1 (PD‐L1) in thymic carcinoma (TC). We also investigated the relationships between these immune checkpoint proteins and the EMT status and examined the impact of induction chemotherapy on patients with tumors that express these proteins.

**Methods:**

The relationship between PD‐1 + TILs/PD‐L1 and clinicopathological findings including EMT was investigated by immunohistochemistry (IHC) of surgically resected samples from 43 patients with TC. In 15 patients receiving induction therapy (IT), those factors were compared before and after IT.

**Results:**

With IHC, 26 cases (60.5%) were positive for PD‐L1, and 19 cases were positive for PD‐1 + TILs (44.2%). The disease‐free survival rate in patients showing EMT and who were PD‐1/PD‐L1 positive was significantly worse compared to negative cases (EMT; *P* = 0.0095, PD‐1; *P* = 0.001, PD‐L1; *P* = 0.0037). We found a significant relationship between PD‐L1 and EMT status (*P* = 0.01). In patients who received IT, PD‐L1 increased, and the change was strongly correlated with EMT status (*P* = 0.01).

**Conclusion:**

Epithelial‐mesenchymal transition, PD‐L1, and PD‐1 + TILs have prognostic impact, and PD‐L1 is correlated with EMT status. PD‐L1 expression after IT was significantly higher compared to before IT and was correlated with the EMT change. Thus, PD‐L1 may be upregulated during EMT, and anti‐PD‐1/PD‐L1 immunotherapy may provide reliable treatment of TC in combination with chemotherapy.

## 1 INTRODUCTION

Thymic carcinoma (TC) is an aggressive thoracic malignancy. Because of the small number of cases, the biological and oncological characteristics of TC are poorly understood, and no standard treatment strategy has been established. Surgery, chemotherapy, and radiation therapy are performed to treat TC. Surgery is often the first treatment in clinical practice when complete resection appears possible according to preoperative imaging. However, tumor invasion of surrounding tissues and regional lymph node metastasis frequently occur. In patients with advanced clinical stages, multimodal therapy such as induction chemotherapy or chemoradiotherapy (followed by surgery) is performed. Despite those treatments, the recurrence rate and mortality rate remain high.

The pathway involving the immune checkpoint proteins, programmed cell death 1 (PD‐1) and PD‐1 ligand 1 (PD‐L1), plays an important role in cancer progression and the cancer microenvironment.^1^, ^2^ Immunotherapies targeting these molecules have been developed and have shown promising results against several malignancies in clinical trials.^3^, ^4^, ^5^ The expression of PD‐L1 in cancer cells and the presence of PD‐1‐positive tumor‐infiltrating lymphocytes (PD‐1 + TILs) are also useful prognostic factors for the therapeutic effect of such immunotherapies in several malignancies.^3^, ^4^, ^5^, ^6^ However, the clinical significance of PD‐1 + TILs and PD‐L1 in TC remains unclear. Moreover, little is known about the mechanism of PD‐L1 expression in TC.

Epithelial‐mesenchymal transition (EMT) is a key process in cancer progression and distant metastasis inseveral malignancies including non‐small cell lung cancer (NSCLC).^7^, ^8^ Transforming growth factor‐β1 (TGF‐β1), fibroblast growth factor, and several other signaling pathways induce EMT, resulting in alterations in EMT‐associated markers including downregulation of E‐cadherin (E‐cad) and upregulation of N‐cadherin (N‐cad) in cancer cells. The above EMT markers are useful prognostic biomarkers.^9^ In addition, chemotherapy and chemo‐resistance induce EMT.^7^ During EMT, loss of cell‐cell adhesion occurs, and cancer cells acquire metastatic potential. Recently, several groups reported a significant relationship between PD‐L1 expression and EMT status in NSCLC.^10^, ^11^, ^12^ Using an in vitro experiment, our group also showed that TGF‐β1 and chemo treatment enhance PD‐L1 expression. Moreover, using immunohistochemistry (IHC) analysis of surgically resected NSCLC samples before and after IT, we also showed that PD‐L1 expression is correlated with EMT status.^12^ However, the role EMT plays in TC progression and the relationship between PD‐L1 expression and clinicopathological findings is unclear in TC. The aim of the present study was to examine the clinical significance of EMT and immune checkpoint proteins in TC progression and the impact of anticancer agents on their expression. We also examined the relationships between expression of these immune checkpoint proteins (PD‐1/PD‐L1) and the clinical background including EMT markers using IHC staining of TC clinical samples obtained by surgical resection.

## 2 PATIENTS AND METHODS

### 2.1 Study population

This study was a retrospective analysis of 43 patients with TC whose tumors were completely resected between 1996 and 2017 at Osaka University Hospital (Osaka, Japan). The Institutional Review Board approved this study, and written informed consent for the study and surgery was obtained from each patient. A computed tomography (CT) scan was routinely performed every 6 months as follow‐up.

### 2.2 IHC staining analysis of EMT markers and PD‐1/PD‐L1 expression in clinical samples

Using clinical samples obtained from enrolled patients with TC, we performed IHC with several antibodies that recognize cancer‐associated proteins, including c‐kit (TC marker), PD‐1/PD‐L1 (immune checkpoint proteins), and E‐cad, N‐cad, and TGF‐β (EMT‐associated proteins). The primary antibodies used in this study are listed in the Data S1. E‐cad, N‐cad, and TGF‐β were used for EMT evaluations. In 15 cases, we were able to compare IHC results before and after IT (samples were obtained by needle biopsy before IT; samples were obtained by surgical resection after IT). For patients who underwent surgery alone, we analyzed the IHC results obtained during surgical resection.

Immunohistochemistry staining for EMT evaluations was performed as in previous reports.^12^ Formalin‐fixed paraffin‐embedded tissue sections were deparaffinized and rehydrated. For staining, we used an automated staining instrument, Histostainer® (Nichirei Biosciences). For antigen retrieval, the sections were boiled in 1 mmol/L EDTA, pH 8.0, and then incubated for 15 minutes at a sub‐boiling temperature. EMT status was evaluated according to N‐cad, E‐cad, TGF‐β, and vimentin staining intensities. In brief, the stained specimens were scored in a semi‐quantitative manner by assessing the staining percentage (0%‐100%) and the intensity (0 = no staining, +1 = weak staining, +2 = distinct staining, +3 = very strong staining). The H score was calculated by multiplying the percentage by the intensity. The H score was classified as 0 (score <10), +1 (≥10 and <30), or +2 (≥30 and <70), or +3 (≥70). We defined EMT‐positive samples as those with N‐cad high‐intensity staining with a score of 2 or 3 and E‐cad low‐intensity staining with a score of 0 or 1. For patients who underwent IT followed by surgery, we also compared expression changes in EMT markers before and after IT. We defined the EMT change‐positive samples as those with the E‐cad H score decreased and the N‐cad H score increased before IT compared to after.

PD‐L1 expression was evaluated as the percentage of cells staining positive for PD‐L1 (tumor proportion score, TPS), and PD‐L1 positive was defined as TPS ≥50%. In addition, we defined cases with an increase in PD‐L1 TPS after IT as PD‐L1 change‐positive by comparing PD‐L1 IHC results before and after IT. PD‐1 IHC was evaluated as the extent of TILs after hematoxylin‐eosin staining and PD‐1‐positive TILs (PD‐1 + TILs) using a visually estimated four‐point scale: 0 (absent), 1 (<30%), 2 (30%‐60%), and 3 (>60%). Samples with a score of 0 or 1 were considered negative, and those with a score of 2 or 3 were considered positive.

### 2.3 Statistical design and data analysis

The correlations between clinicopathological factors and variables (PD‐L1, PD‐1, and EMT status) were evaluated by Fisher's exact test. Categorical variables including sex, Masaoka stage, and histopathology and continuous variables including tumor size were evaluated with analysis of variance (ANOVA). The histological response to induction therapy was classified into four categories as follows: Ef0: no histological response, Ef1: more than one‐third of the tumor cells viable, Ef2: less than one‐third of the tumor cells viable, Ef3: no viable tumor cells according to General Rule for Clinical and Pathological Record of Lung Cancer.^13^ The response to the induction therapy was evaluated by CT scan based on the RECIST guideline (version 1.1).^14^

Disease‐free survival (DFS) and overall survival (OS) were analyzed with the Kaplan‐Meier method, and the log‐rank test was used to compare the survival distributions of subgroups. DFS was defined as the time from the data of surgery to the first event of either disease recurrence or death due to any cause. OS was defined as the time from the date of surgery to death due to any cause. The correlation between PD‐L1 IHC status (TPS) and variance was analyzed with ANOVA. The univariate Cox proportional hazards regression model was used to analyze DFS. All statistical analyses were performed with JMP version 13 for Windows (SAS Institute Inc, Cary, NC, USA). *P* values <0.05 were considered to be statistically significant.

## 3 RESULTS

### 3.1 Patient cohorts

The median follow‐up time was 4.3 years (95% confidence interval (CI): 3.0‐5.5 years). Patient characteristics are listed in Table 1. The patients comprised 31 males and 12 females with an average age of 59.9 years (95% CI: 55.7‐64.1 years). Of the 43 total patients, 23 (53.5%) underwent IT followed by surgery; induction chemoradiation therapy was performed in 16 patients, and induction chemotherapy was performed in seven patients. Initial surgery was performed in 20 patients (46.5%). All chemotherapy regimens were platinum‐based chemotherapy (Table S1). Four weeks after IT, surgery was performed. The average tumor size was 57.0 mm (95% CI: 50.1‐63.9 mm). Regarding Masaoka stage, four patients were stage I, one was stage II, 23 were stage III, five were stage IVa, and 10 were stage IVb. Invasion into surrounding tissues was found in 38 cases (88.4%), and combined resection of those invasive tissues was performed. In all cases, tumor resection and total thymectomy were performed through the median sternotomy. In the cases with surrounding organs invasion, combined resection of invaded organs was carried out. In 38 cases (88%), combined resections were perfomed; veins including the superior vena cava in 22 (57.9%), lung resection in 23(53.4%), and arteries including the aortic arch in 4(9.3%; there was some overlap; Table S2).

**Table 1:** Patient** characteristics**

Variable	PD‐L1−	PD‐L1＋	*P*	PD‐1 − TILs	PD‐1 + TILs	*P*	EMT‐	EMT+	*P*	(%)
Number of patients (%)	26 (60.5)	17 (39.5)		24 (55.8)	19 (44.2)		8 (18.6)	35 (81.4)		43
Gender										
Male										26 (72.2)
Female										10 (27.8)
Tumor size (mean ± SD, mm)	59.2 ± 23.5	53.7 ± 10.7	0.4	59.3 ± 25.1	63.8 ± 31.0	0.4	63.8 ± 31.0	55.5 ± 20.2	0.3	57 ± 22.4
Masaoka stage										
I	3	1	0.69	3	1	0.33	0	4	0.64	4
II	1	0		0	1		0	1		1
III	15	8		14	9		5	18		23
IVa	2	3		1	4		0	5		5
IVb	5	5		6	4		3	7		10
Combined resection number (%)										
Done	23	15	1.0	21	17	1.0	8	30	0.56	38 (88.4)
Not done	3	2		3	2		0	5		5 (11.6)
Induction therapy number (%)										
Done	10	13	0.02	14	9	0.54	5	18	0.70	23 (53.5)
Not done	16	4		10	10		3	17		20 (46.5)
Histopathology number (%)										
Squamous	18	14	0.48	15	17	0.07	5	27	0.4	32 (74.4)
Others	8	3		9	2		3	8		11 (25.6)
Response rate^a^ (RECIST) N = 23, number (%)										
CR	0	0	0.02	0	0	1.0	0	0	0.02	0 (0)
PR	4	0		3	1		3	1		4 (17.4)
SD	6	13		11	8		2	17		19 (82.6)
PD	0	0		0	0		0	0		0 (0)
Histopathological response^b^ N = 23 number (%)										
Ef 1	3	7	0.4	5	5	0.41	0	10	0.04	10 (43.5)
Ef 2/3	7	6		9	4		5	8		13 (56.5)

a Response rate; CR; complete response, PR; partial response, SD; stable disease, PD; progressive disease according to the Response Evaluation Criteria in Solid Tumors (RECIST).

b Histopathological response; EF, histopathological response effect using General Rule for Clinical and Pathological Record of Lung Cancer; Ef1, some necrosis of tumor cells with more than one‐third of tumor cells were viable; Ef2, less than one‐third of tumor cells were viable; Ef3, no tumor cells were viable.

### 3.2 Clinical impact of the expression of PD‐L1 and the presence of PD‐1 + TILs in TC

**Figure 1:**
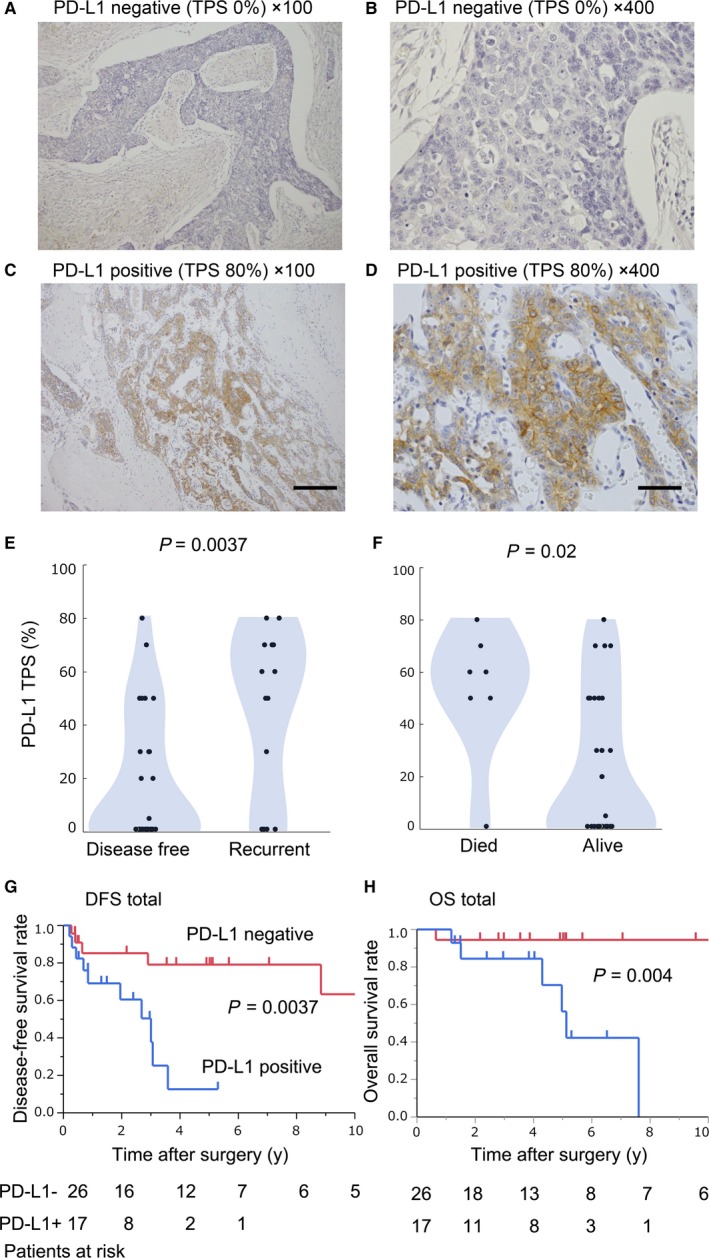
Immunohistochemical staining of PD‐L1 and the clinical impact. Representative images of IHC staining for PD‐L1 in a resected tumor from a patient with thymic carcinoma. (A‐B) PD‐L1‐negative staining (Tumor proportion score; TPS 0% A; ×100, B; ×400) and PD‐L1‐positive staining (TPS 80%, C; ×100, D; ×400). Scale bars (×100); 200 µm, Scale bar (×400); 50 µm. Panels E and F show dot plots depicting PD‐L1 TPS according to the clinical outcome. Panels G and H show disease‐free survival (DFS) and overall survival (OS) based on PD‐L1 status (G; DFS, *P* = 0.0037, H; OS, *P* = 0.04)

We analyzed the clinical implication of PD‐L1 expression in TC. Figure 1A‐D show representative images of PD‐L1 IHC staining of surgically resected samples. Figure 1A,B show typical PD‐L1‐negative images (TPS 0%). Figure 1C,D show typical PD‐L1‐positive images (TPS 80%). Seventeen cases (39.5%) showed over 50% TPS following PD‐L1 IHC. Recurrent and fatal cases showed significantly higher PD‐L1 TPS compared to that of disease‐free and surviving patients (Figure 1E; *P* = 0.0037, and Figure 1F; *P* = 0.02). In addition, Kaplan‐Meier analysis showed that PD‐L1‐positive TPS patients had a significantly worse DFS rate compared to PD‐L1‐negative patients (Figure 1G; *P* = 0.0037). A significant relationship between PD‐L1 expression and OS was also found (Figure 1H; *P* = 0.004). The relationships between PD‐L1 expression and clinicopathological factors are shown in Table 1. We found no significant relationships between the PD‐L1 expression level and tumor size, histopathological analysis, or Masaoka stage. While there were significant relationship between PD‐L1 expression level and administration of IT, univariate DFS Cox analyses showed that PD‐L1 positivity had a prognostic value (Table 2).

**Table 2:** Univariate analysis of disease‐free survival according to selected clinical factors

Factor	Hazard Ratio	95% CI	*P*
Sex			
Female	1		
Male	1.43	0.48‐5.20	0.53
PD‐1			
Negative	1		
Positive	4.19	1.46‐13.65	0.0076
Age (y)			
<70	1		
≥70	0.65	0.15‐2.07	0.49
Masaoka stage			
I	1		
III	5.60E+8	0.69‐	0.0046
IVb	1.90 E + 9	2.4‐	0.09
PD‐L1 TPS (%)			
≥50	1		
<50	5.03	1.62‐18.9	0.0046
EMT			
Negative	1		
Positive	2.6E+9	3.31‐3.31	0.0009

**Figure 2:**
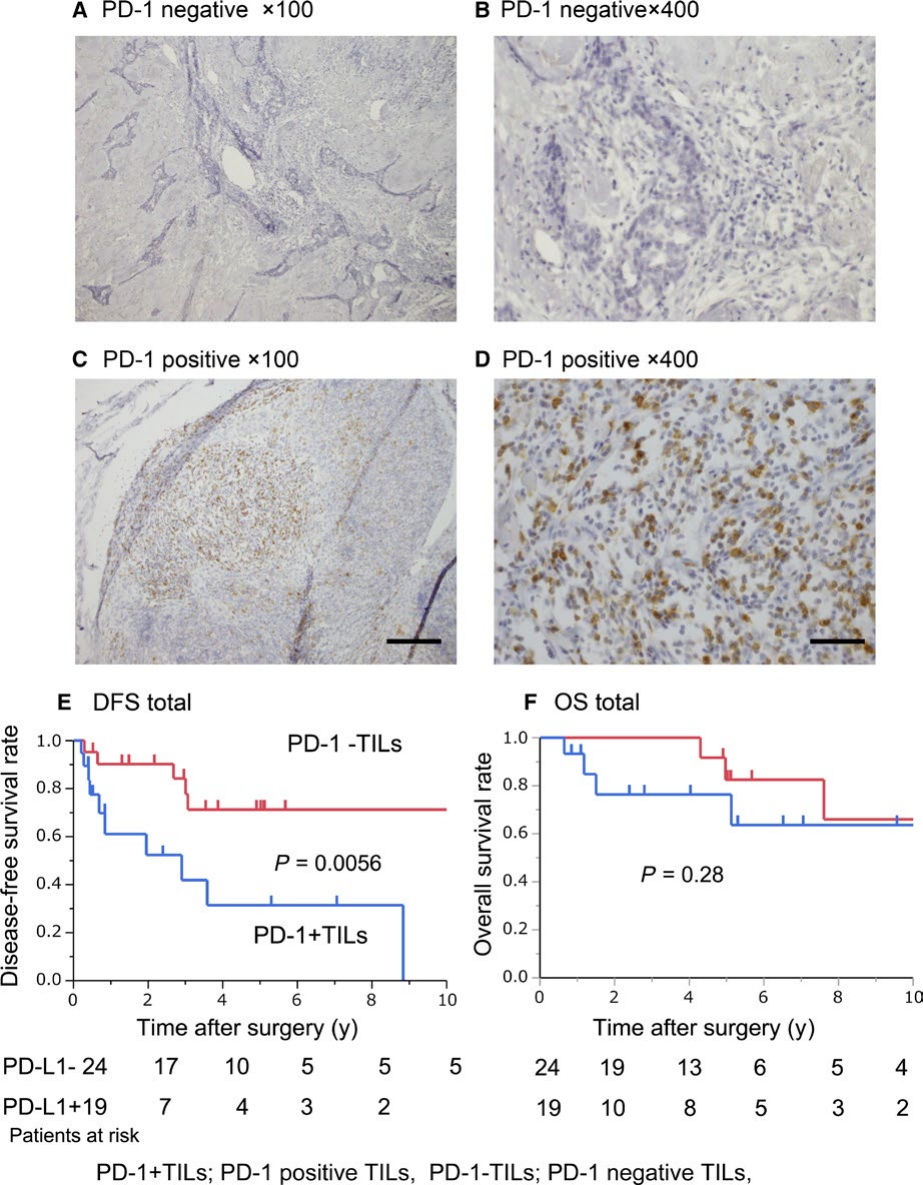
Immunohistochemical staining of PD‐1 in TILs and the clinical impact. Representative images of IHC staining for PD‐1 in tumor‐infiltrating lymphocytes (TILs) in resected tumors from patients with thymic carcinoma. (A‐B) PD‐1 − TILs (PD‐1 − TILs; A; ×100, B; ×400) and PD‐1 + TILs (PD‐1 + TILs; C; ×100, D; ×400). Scale bars (×100); 200 µm, Scale bar (×400); 50 µm. Disease‐free survival (DFS) and overall survival (OS) were evaluated based on PD‐1 TIL status (E; DFS, *P* = 0.0056, F; OS, *P* = 0.28). PD‐1 − TILs and PD‐1 + TILs mean PD‐1‐negative and PD‐1‐positive TILs, respectively

Next, we analyzed the clinical implications of PD‐1 + TILs. Figure 2A,B show typical IHC images of surgically resected specimens with PD‐1‐negative TILs (PD‐1 − TILs). Figure 2C,D show typical IHC images of PD‐1 + TILs. Nineteen of 43 cases (44.2%) had PD‐1 + TILs. The relationships between the presence of PD‐1 + TILs and the clinicopathological background are shown in Table 1. We found no significant relationship between the presence of PD‐1 + TILs and Masaoka stage, tumor size, or the administration of IT. Kaplan‐Meier analysis showed that the DFS rate in patients with PD‐1 + TILs was significantly worse compared to patients with PD‐1‐TILs (Figure 2E; *P* = 0.0056). We found no significant relationship for OS regarding PD‐1 expression by TILs (Figure 2F; *P* = 0.28). Univariate Cox proportional hazards regression models for estimating DFS showed that the presence of PD‐1 + TILs was one of the prognostic factors (Table 2).

### 3.3 The clinical impact of the EMT status and its relationships with PD‐L1 expression in TC

**Figure 3:**
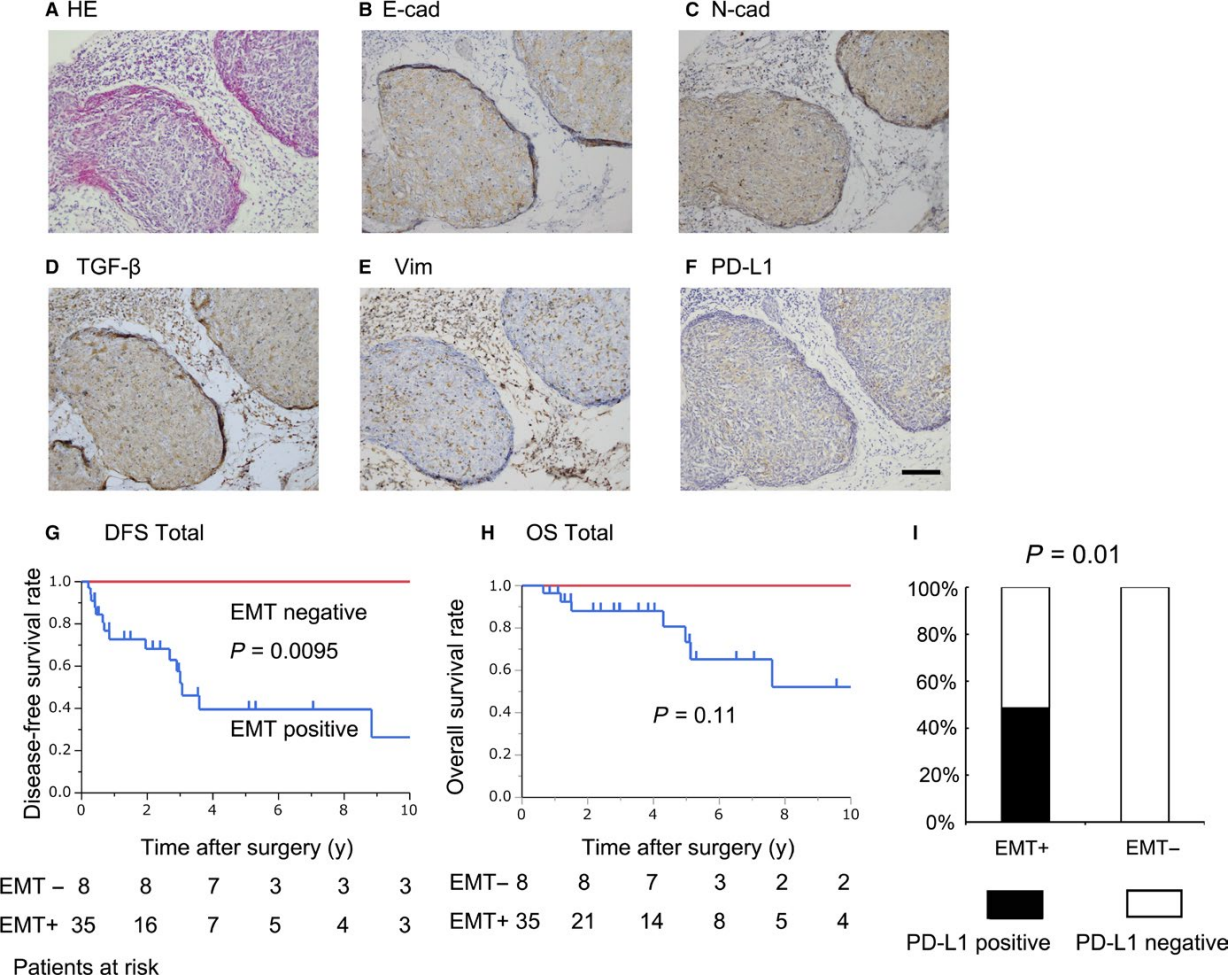
Immunohistochemical staining of the EMT status, its clinical impact, and its relationship with the PD‐L1 status. Representative images of positive IHC staining of EMT markers and PD‐1/PD‐L1 in thymic carcinoma. (A) HE; hematoxylin‐eosin stain, (B) E‐cad; E‐cadherin, (C) N‐cad; N‐cadherin, (D) TGF‐β, (E) Vim; vimentin, (F) PD‐L1. Scale bars (×100); 200 µm. Disease‐free survival (DFS) and overall survival (OS) were evaluated based on EMT status (G; DFS, *P* = 0.0095, H; OS, *P* = 0.11). EMT−and EMT+ mean EMT‐negative and EMT‐positive, respectively. The relationships between EMT status and PD‐L1 (I; *P* = 0.01). EMT+; EMT positive, EMT‐; EMT negative

The clinical significance of the EMT status in TC remains unclear. Here, we investigated the clinical implication of EMT as a prognostic factor in TC and examined the relationship between EMT and clinical backgrounds including PD‐L1 expression with IHC staining of TC specimens. The relationships between the presence of EMT status and the clinicopathological background are shown in Table 1. We found no significant relationship between the EMT‐positive status and Masaoka stage, tumor size, or the administration of IT while there were significant relationships between EMT status and histopathological response effect after IT. Figure 3A‐F show representative images of an EMT and PD‐L1‐positive TC case. Figure 3A shows a typical TC specimen stained with hematoxylin‐eosin. In addition, the weak E‐cad expression and strong N‐cad expression are shown in Figure 3B,C, respectively. Moreover, TGF‐β and vimentin IHC also showed positive expression (Figure 3D,E). EMT‐positive patients showed significantly worse DFS compared to EMT‐negative patients (Figure 3G; *P* = 0.0095). No significant relationship was found between EMT‐positive and EMT‐negative patients for OS (Figure 3H; *P* = 0.11). Univariate analyses of DFS also showed that positive EMT status was a prognostic factor (Table 2, *P* < 0.01). These results suggest that EMT is a potential prognostic factor for recurrence after surgical resection of TC.

Figure 3I shows that PD‐L1‐positive cases had a significantly higher EMT‐positive rate compared to EMT‐negative cases (*P* = 0.002).

### 3.4 Upregulation of PD‐L1 TPS and alteration in EMT markers after IT

**Figure 4:**
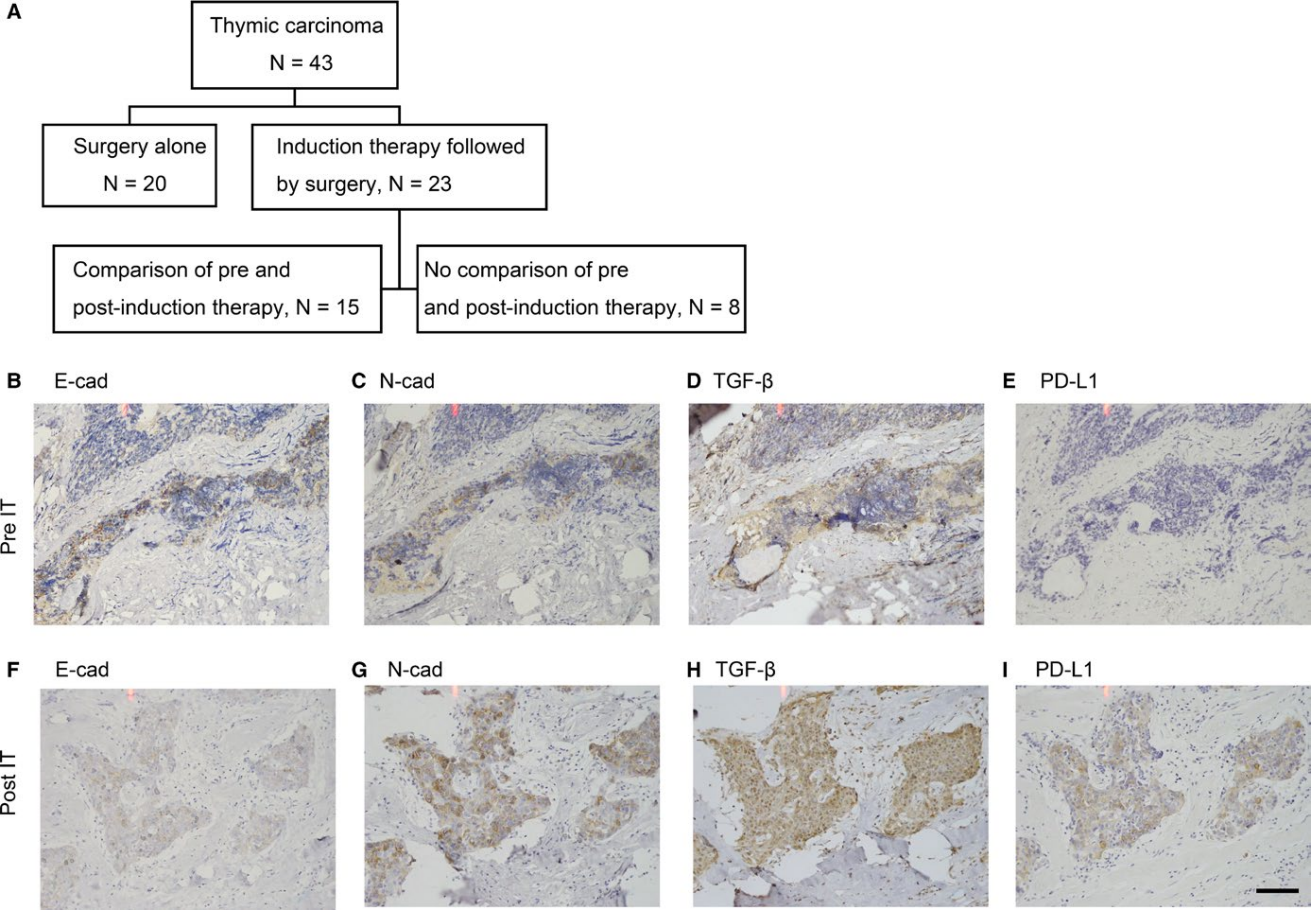
Immunohistochemical staining of a clinical sample before and after induction therapy. Panel A shows the flow chart of enrolled patients. Representative images of IHC in samples obtained before and after induction therapy. (B‐E) IHC results of a clinical sample obtained with CT‐guided percutaneous biopsy before IT. (F‐I) IHC results of a surgically resected sample after induction therapy. (B, F) E‐cad, (C, G) N‐cad, (D, H) TGF‐β, (E, I) PD‐L1. Scale bars (×100); 200 µm

Next, to explore whether these relationships between PD‐L1 expression and EMT status after IT in TC are similar to our previous report of NSCLC,^12^ we performed IHC staining of those molecules in 15 comparable cases who underwent IT followed by surgery (Figure 4A). A total of 8 cases were excluded due to obtaining no available samples before IT. Figure 4B‐I shows representative IHC images of EMT‐associated markers and PD‐L1 before and after IT. Figure 4B‐E show IHC images of clinical samples obtained by CT‐guided needle biopsy before IT, and Figure 4F‐I show a clinical sample obtained by surgical resection after IT in the same patient. The intensity of E‐cad after IT decreased compared to that before IT (Figure 4B vs F). Postoperative N‐cad and TGF‐β intensity increased compared to preoperative images (N‐cad; Figure 4C vs G, TGF‐β; Figure 4D vs H). PD‐L1 staining intensity after IT also increased compared to the intensity before IT (Figure 4E vs I).

**Figure 5:**
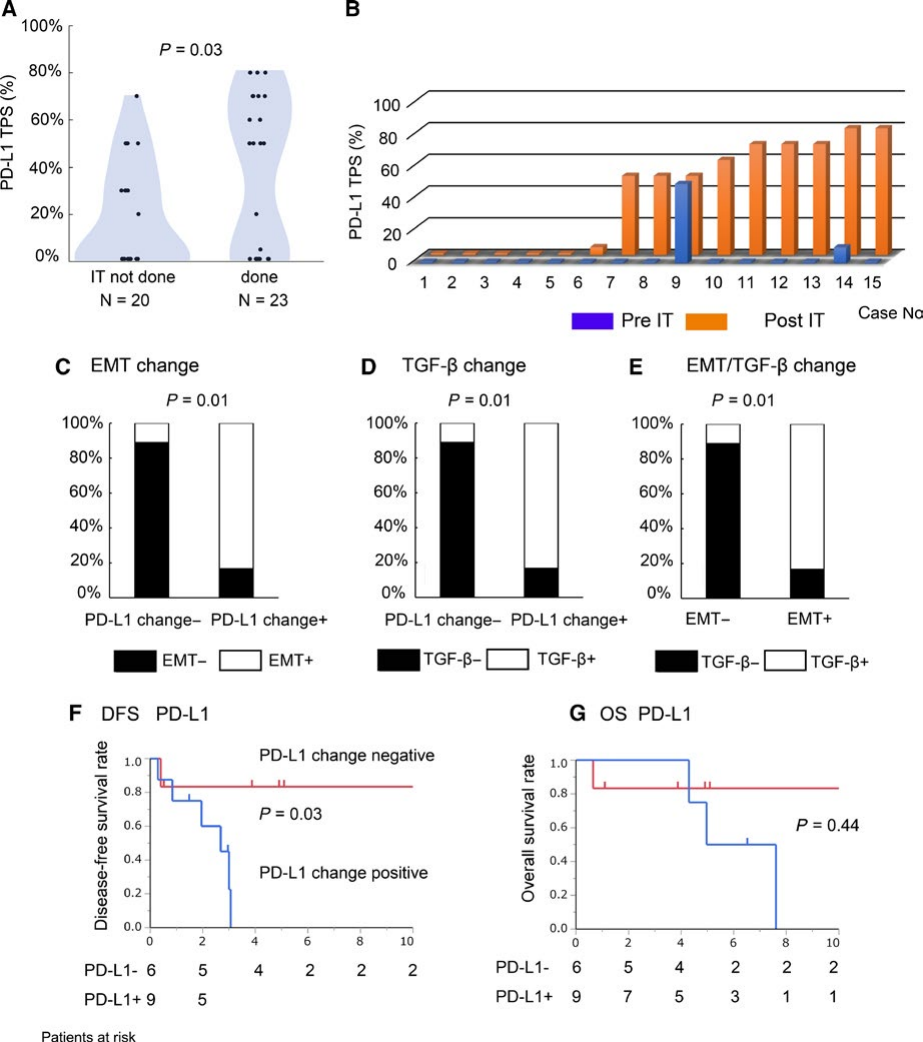
The relationships between PD‐1/PD‐L1 expression and EMT status with or without induction therapy (IT) and clinical impact of the presence of PD‐L1 change. Panel A shows a dot plot depicting PD‐L1 TPS according to induction therapy (IT) (*P* = 0.03). Panel B shows PD‐L1 TPS of each case before and after IT. Panels C‐E show the relationships between the two factors shown as analyzed in 15 patients who underwent IT followed by surgery. The relationships between EMT and PD‐L1 change (C; *P* = 0.01), between TGF‐β and PD‐L1 change (D; *P* = 0.01), and between EMT and TGF‐β change (E; *P* = 0.01) are shown. Panels f and g show Kaplan‐Meier survival curves according to the presence of PD‐L1 change (F; DFS, *P* = 0.03, G; OS, *P* = 0.44)

The PD‐L1 TPS of patients who underwent IT was significantly higher compared with the TPS of patients who did not undergo IT (Figure 5A). Next, we compared PD‐L1 TPS before and after IT in patients who also underwent surgery (Figure 5B; N = 15 comparable cases of total 23 cases who underwent IT followed by surgery). Of the 15 cases, nine cases showed increased PD‐L1 TPS after IT, while six cases showed no change of PD‐L1 TPS (Figure 5B). The Wilcoxon signed rank test indicated that PD‐L1 TPS after IT was significantly higher than before IT (Figure 5B; *P* = 0.004). We also evaluated IHC staining of PD‐L1 and EMT markers including TGF‐β before and after IT in the same patients and compared their status before and after IT. Upregulation of PD‐L1 TPS (PD‐L1 change positive; PD‐L1 change+) showed a significant correlation with EMT positivity and with a change in TGF‐β (Figures 5C,D; *P* = 0.0035). In addition, we found a significant relationship between the positive rate of EMT and the TGF‐β change (Figure 5E; *P* = 0.0035).

### 3.5 PD‐L1 TPS upregulation after IT resulted in a poor prognosis

Next, we examined the prognostic impact of the presence of a change in PD‐L1 TPS in 15 patients who underwent IT followed by surgery. Figure 5F,G shows Kaplan‐Meier analysis of DFS and OS according to changes in PD‐L1 TPS. Regarding DFS, a positive change in PD‐L1 TPS was associated with a significantly higher recurrence rate compared to a negative change (Figure 5F; *P* = 0.03). Regarding OS, we found no significant relationship between the presence or absence of a change in PD‐L1 (Figure 5F; *P* = 0.44).

## 4 DISCUSSION

We analyzed IHC staining of EMT markers and the immune checkpoint proteins, PD‐1 and PD‐L1, in clinical samples of TC obtained by surgical resection. Our data suggest that the EMT status and immune checkpoint proteins have prognostic impact and that their expression is closely related. To our knowledge, our study analyzed one of the largest number of TC surgical samples regarding EMT status and PD‐1/PD‐L1 expression.

To overcome advanced TC, surgery and chemotherapy are the maintherapeutic options. Despite those multimodal therapies, the outcome is far from satisfactory, and no standard therapy has been established. One reason is that biological research about TC has not made much progress compared to other carcinomas. Because TC is rare, our clinical experience has been insufficient, and oncological characteristics are poorly understood. In the present study, we revealed the microenvironment of TC from the viewpoint of cancer immunology. Recently, immunotherapy has been developed as another option for several malignancies. Agents that block PD‐1/PD‐L1 have exhibited dramatic antitumor efficacy in clinical trials for patients with a variety of cancer types.^3^, ^4^, ^5^ PD‐1 and its ligand, PD‐L1, play a major role in the cancer microenvironment, and expression of these molecules is related to not only prognosis but is also a useful predictive factor for the therapeutic effect of anti‐PD‐1 or anti‐PD‐L1 immunotherapies in various malignancies.^6^

However, the clinical significance of PD‐L1 in TC is poorly understood. Although a few groups have discussed the clinical significance of PD‐1/PD‐L1 expression in TC with IHC analysis, the significance as a predictive factor is controversial. Katura et al showed that high PD‐L1 expression is correlated with unfavorable prognosis,^15^, ^16^ whereas Yokoyama et al^17^ reported that low PD‐L1 expression is related to favorable prognosis. In our data, high PD‐L1 expression and the presence of PD‐1 + TILs were seen in patients with worse prognosis, similar to other malignancies. We also previously reported the same results in which PD‐L1 expression has predictive value in NSCLC.^12^ Those discrepancies may be due to differences in clinical backgrounds and the numbers of samples. Regarding the first point, former reports included PD‐L1 IHC results from not only samples obtained from surgical resection but also small specimens obtained by needle biopsy.^15^, ^16^ In contrast, we performed IHC staining for PD‐L1 and other predictive markers using only surgically resected samples. Ilie et al^18^ indicated a discrepancy in IHC evaluation of PD‐L1 expression between biopsy samples and surgically resected samples. Another difference is in the IHC cutoff score and the antibodies used for IHC. Several antibodies for PD‐L1 assays are commercially available. Sakane et al and other groups showed differences and concordance between antibodies in terms of IHC intensity and the cutoff point in TC.^19^, ^20^, ^21^ A third difference is whether the sample used for IHC staining was obtained before or after IT. The former groups disregard the effect of IT in their PD‐L1 IHC results. However, other previous reports including ours showed that chemotherapy enhances PD‐L1 expression in NSCLC.^12^, ^22^, ^23^ In the present study, we showed the same results in TC; PD‐L1 TPS after IT was significantly higher compared to PD‐L1 TPS before IT.

Epithelial‐mesenchymal transition plays a crucial role in cancer progression, and this phenomenon can be found in the cancer microenvironment in several types of cancers. EMT is promoted by TGF‐β secreted from not only the cancer cell itself but also from other cell types. EMT confers invasive and metastatic abilities on cancer cells that are necessary for metastasis.^7^, ^8^, ^9^ However, the role of EMT in TC progression remains unclear. The most common type of TC is squamous cell carcinoma, and the clinical implications of EMT in squamous cell carcinoma from other origins, such as the neck, ovary, and esophagus, have been reported by several groups.^24^ Tsutsumi et al^25^ showed that the EMT status has predictive value and enhances malignancy including metastasis and invasion in esophageal squamous cell carcinoma. Our data also showed the same results in which EMT markers are candidate prognostic factors in TC. This is the first report to discuss the clinical significance of EMT in TC. Moreover, our results suggest that EMT status is strongly related with the PD‐L1 expression in TC. In other malignancies such as NSCLC, several groups and our group previously reported a significant relationship between the EMT status and PD‐L1 expression.^12^, ^24^, ^25^ PD‐L1 expression is regulated by the TGF‐β pathway and the EMT process. Moreover, EMT is regulated by several stimuli. In recent studies, chemotherapy was reported to enhance EMT.^7^ Zhang et al demonstrated that chemo‐preventive agents induce PD‐L1 in human breast cancer cells and promote PD‐L1‐mediated interferon‐γ and T‐cell apoptosis.^11^, ^22^ Hecht et al^23^ also showed that PD‐L1 expression in rectal adenocarcinoma is upregulated after chemoradiotherapy compared with before.

We hypothesized that PD‐L1, N‐cad and TGF‐β expression was also upregulated after chemotherapy induction in TC. To test this hypothesis, we performed IHC for PD‐L1 and EMT markers including TGF‐β and compared the results before and after IT. Similar to our previous reports regarding NSCLC,^12^, ^26^ we obtained the same results for TC in which PD‐L1 expression was correlated with an EMT change and TGF‐β expression change after chemotherapy. Those results suggest that chemotherapy enhanced PD‐L1 expression through the chemo‐induced TGF‐β signaling pathway in TC. The regulation of PD‐L1 expression by EMT and the TGF‐β pathway may be a fundamental phenomenon in cancer immunology.

In general use of immune‐check point inhibitor (ICI), PD‐L1 IHC assay is used and the results are biomarker for patient selection and therapeutic response of immune‐check point inhibitor (ICI). However, there are several problems such as sensitivity and appropriate cutoff in the IHC assays. From our results, EMT markers may be useful surrogate markers for case selection of ICI after anticancer agent.^19^

Our study has some limitations. The first limitation is the small sample size. Therefore, the predictive role of PD‐L1 expression is still controversial. Future studies should involve increased numbers of cases in collaboration with multiple institutions. As a second limitation, our IHC data provide supporting evidence that PD‐L1 expression is enhanced thorough the EMT process or TGF‐β signaling in TC. The mechanism of regulation of PD‐L1 expression needs to be elucidated by utilizing molecular biological techniques, although there are currently no commercially available TC cell lines.

In conclusion, understanding the mechanism of PD‐L1 expression will yield important information regarding the role of immune checkpoint proteins in TC. Our results suggest that EMT markers may be informative for making decisions about induction of immunotherapy. In addition, our results suggest that the EMT status plays an important role in cancer progression and has a significant relationship with PD‐L1 expression. Our results also show that PD‐1/PD‐L1 blockade after chemotherapy or in combination with chemotherapy could be more effective than monotherapy and suggest another therapeutic option for treating chemo‐resistant cancer.

## CONFLICT OF INTEREST

The authors have declared no conflicts of interest. We state that the material has not been previously published or submitted elsewhere for publication. All authors have read and approved the manuscript and made a sufficient contribution to the work. We will transfer copyright to the Publisher.
