# Supplementary material for: The prognostic impact of programmed cell death 1 and its ligand and the correlation with epithelial‐mesenchymal transition in thymic carcinoma
Source: Cancer Med. 2019 Jan 1;8(1):216–26. doi: 10.1002/cam4.1943 (PMC6346217; doi:10.1002/cam4.1943)
Supplement: Supplementary file 1 [file CAM4-8-216-s001.docx]

Data S1

The antibodies used for IHC were as follows:

monoclonal mouse anti-human N-cadherin (6G11/M3613, 1/50; DAKO®, Denmark), monoclonal mouse anti-human E-cadherin (NCH-38/M3612; DAKO®),

polyclonal rabbit anti-human TGF-β (ab66043; Abcam®, UK),

rabbit monoclonal anti-human PD-L1 (clone SP142, 1/100; Spring Bioscience®, USA), mouse monoclonal anti-human PD-1 (NAT105, 1/50 ab52587, Abcam®), and

rabbit monoclonal anti-human vimentin (Code 412551, HISTOFINE®, Japan).

Table S1

Chemotherapy regimen

N=23

| CODE | 1 |
| --- | --- |
| ADOC | 1 |
| TJ | 9 |
| CDDP+TXT | 9 |
| CDDP+5FU+TXT | 1 |
| CDDP+PTX | 1 |
| CDDP+VP16 | 1 |

CODE; Cyclophosphamide, Vincristine, Doxorubicin and Etoposide

ADOC; Adriamycin, Cisplatin, Vincristine and Cyclophosphamide

CDDP; Cisplatin

CBDCA; Carboplatin

PTX; Paclitaxel

TXT; Docetaxel

VP16; Etoposide

TJ; paclitaxel and carboplatin

Table S2

Combined resection organs

Organs N=38

| A | 1 |
| --- | --- |
| A+V | 2 |
| A+V+N | 1 |
| Lung | 7 |
| Lung+A | 1 |
| Lung+N | 3 |
| Lung+Peri | 2 |
| Lung+V | 4 |
| Lung+V+N | 5 |
| Lung+V+Peri | 1 |
| Peri | 2 |
| V | 6 |
| V+N | 3 |

V; vein including brachiocephalic vein or super vena cava, A; artery including brachiocephalic artery or aortic arch, Peri; pericardium, N; nerve including phrenic nerve or vagus nerve.
